# Alterations in the gut microbiome and metabolome profiles of septic mice treated with Shen FuHuang formula

**DOI:** 10.3389/fmicb.2023.1111962

**Published:** 2023-03-08

**Authors:** Shasha He, Chunxia Zhao, Yuhong Guo, Jingxia Zhao, Xiaolong Xu, Yahui Hu, Bo Lian, Haoran Ye, Ning Wang, Lianxiang Luo, Qingquan Liu

**Affiliations:** ^1^Beijing Hospital of Traditional Chinese Medicine, Capital Medical University, Beijing, China; ^2^Beijing Institute of Chinese Medicine, Beijing, China; ^3^Beijing Key Laboratory of Basic Research with Traditional Chinese Medicine on Infectious Diseases, Beijing, China; ^4^Tianjin University of Traditional Chinese Medicine, Tianjin, China; ^5^Beijing Chaoyang Hospital, Capital Medical University, Beijing, China; ^6^The Marine Biomedical Research Institute, Guangdong Medical University, Zhanjiang, Guangdong, China

**Keywords:** sepsis, traditional Chinese medicine, Shen FuHuang formula, gut microbiome, metabonomics

## Abstract

Sepsis has a high mortality rate, and treating sepsis remains a significant challenge worldwide. In former studies, our group found that traditional Chinese medicine, Shen FuHuang formula (SFH), is a promising medicine in treating coronavirus disease 2019 (COVID-19) patients with the septic syndrome. However, the underlying mechanisms remain elusive. In the present study, we first investigated the therapeutic effects of SFH on septic mice. To investigate the mechanisms of SFH-treated sepsis, we identified the gut microbiome profile and exploited untargeted metabolomics analyses. The results demonstrated that SFH significantly enhanced the mice’s 7-day survival rate and hindered the release of inflammatory mediators, i.e., TNF-α, IL-6, and IL-1β. 16S rDNA sequencing further deciphered that SFH decreased the proportion of *Campylobacterota* and *Proteobacteria* at the phylum level. LEfSe analysis revealed that the treatment of SFH enriched *Blautia* while decreased *Escherichia_Shigella*. Furthermore, serum untargeted metabolomics analysis indicated that SFH could regulate the glucagon signaling pathway, PPAR signaling pathway, galactose metabolism, and pyrimidine metabolism. Finally, we found the relative abundance of *Bacteroides*, *Lachnospiraceae_NK4A136_group*, *Escherichia_Shigella*, *Blautia*, *Ruminococcus*, and *Prevotella* were closely related to the enrichment of the metabolic signaling pathways, including L-tryptophan, uracil, glucuronic acid, protocatechuic acid, and gamma-Glutamylcysteine. In conclusion, our study demonstrated that SFH alleviated sepsis by suppressing the inflammatory response and hence reduced mortality. The mechanism of SFH for treating sepsis may be ascribed to the enrichment of beneficial gut flora and modulation in glucagon signaling pathway, PPAR signaling pathway, galactose metabolism, and pyrimidine metabolism. To sum up, these findings provide a new scientific perspective for the clinical application of SFH in treating sepsis.

## 1. Introduction

Sepsis is defined as life-threatening organ dysfunction caused by a dysregulated host response to infection (Seymour et al., 2016; Shankar-Hari et al., 2016; Singer et al., 2016). Despite the fact that a global burden of disease study demonstrated the age-standardized incidence of sepsis has decreased by 37.0% and mortality by 52.8% from 1990 to 2017, the incidence and mortality rate remained high. A Lancet report indicated in 2017, the incidence of sepsis reached 48.9 million and caused 11 million sepsis-associated deaths (Rudd et al., 2020). The morbidity and mortality of sepsis are kept at a high level in clinical practice regardless of rigorous studies on the pathophysiology and management of sepsis in recent years (Gotts and Matthay, 2016; Hattori et al., 2017). Therefore, it is essential to explore effective drugs to treat sepsis. Professor Qingquan Liu, a top-tier researcher in treating sepsis using TCM, designed Shen FuHuang formula (SFH), an effective traditional Chinese medicine (TCM) prescription for clinical use.

Consisting of *Panax ginseng C.A.Mey*, *Aconitum carmichaelii Debeaux*, and *Rheum palmatum L.*, SFH has proven clinically effective for sepsis patients in the last two decades. Recently, SFH has also shown promising therapeutic effects in treating septic patients who experience comorbid coronavirus disease 2019 (COVID-19) *via* anti-inflammation and anti-thrombosis (Liu T. et al., 2020). However, the exact mechanisms during SFH treatment remain poorly understood. Normal microbiota represents the frontline defense against pathogens, while gut dysbiosis adds to the probable onset of life-threatening infection and sepsis (Miller et al., 2021). The gut microbiome is thought to be closely linked to the etiology and outcome of sepsis since bacterial species that thrive in the absence of anaerobes, such as *Staphylococcus* species and *Escherichia coli*, translocate and cause bacteremia (Adelman et al., 2020). Metabolomics emerged as a novel approach to exploring disease pathogenesis, diagnosis, and prognosis (Lee and Banerjee, 2020), as well as predicting the mortality of septic patients (Ferrario et al., 2016; Wang et al., 2020). In this study, we utilized a murine sepsis model by operating cecal ligation and puncture (CLP) to investigate the therapeutic effects of SFH. 16S rDNA sequencing and untargeted metabolomics assays were then performed to analyze the intrinsic mechanisms of SFH in treating sepsis.

## 2. Materials and methods

### 2.1. Preparation of SFH

For the preparation of SFH, the ingredients were used as followings: *Panax ginseng C.A.Mey* (30 g), *Rheum palmatum L* (30 g), and *Aconitum carmichaelii Debeaux* (60 g). The drugs were steeped in pure water for 30 min. The *Aconitum carmichaelii Debeaux* was decocted first and the remaining ingredients were added into the decocted *Aconitum carmichaelii Debeaux*. These three drugs were decocted together for 30 min and then collected the filtrate. Repeated the process and collect the filtrate. Finally, mixed the filtrate and concentrated with 1 g of the crude drug/mL (used as the mother liquid). Pure water was used to dilute the mother liquid to 0.5 g/mL (high dosage for the treatment) and 0.1 g/mL (low dosage for the treatment).

The quality control of SFH was achieved through ultra-performance liquid chromatography, tandem mass spectrometry MS/MS, and biosystems QTRAP. Briefly, 4 μL test solution was injected into the Agilent SB-C18 column (2.1 mm) at 40°C × 100 mm, with a flow rate at 0.35 mL/min. Mobile phase A and B were prepared as recommended. Supplied with an electrospray ionization (ESI Turbo) source, the QTRAP appliance was used for positive and negative ionization scanning. The ion spray positive mode voltage was set at 5,500 V and its negative mode was 4,500 V. The ion source gas I (GSI), gas I (GSII), and curtain gas (CUR) are set to 50, 60, and 25.0 psi, and the collision-induced ionization parameter was restricted to high.

### 2.2. Animal experiment

Healthy male SPF C57BL/6J mice (18–22 g, 6 weeks old) were purchased from the Beijing Hua FuKang Biotechnology Co, animal Certificate No. SCXK (Beijing) 2019–0008. The animals were taken for tests under the guideline of animal management with a stable light source (12 h of alternating light) and kept in a stable room temperature and humidity. The animal experiments were approved by the Animal Care and Use Committee of Beijing Institute of Chinese Medicine (approval No. 2021030201). According to the references, the sepsis model was replicated using CLP (Rittirsch et al., 2009). Sham-operated animals were not subjected to cecum ligation and perforation, and other steps were consistent with CLP animals. The mice were injected with 2 mL sterile normal saline after surgery.

After 7 days of adaptive feeding, 100 mice were randomly separated into four groups. (1) Sham-operated group (Sham, *n* = 10), mice underwent sham surgery without supplementary medications. (2) CLP group (CLP, *n* = 30), mice that only underwent CLP surgery. (3) SFH low-dose group (SFH-L, *n* = 30), CLP-mice have given an extra 0.5 mL of SFH low-dose decoction, and finally, the (4) SFH high-dose group (SFH-H, *n* = 30), CLP-mice with 0.5 mL high-dose SFH decoction. Mice were orally administered a day after the operation, while in the rest groups, mice were supplied with an equal volume of saline. The mice were continuously treated with SFH or saline for 3 days. An additional 30 mice per group were used in the survival test to assess the survival rate over 7 days.

### 2.3. Sample collection and preparation

At 72 h after CLP operation, sodium pentobarbital (50 mg/kg) was used for anesthetization. For serum collection, a syringe was used to obtain blood from the heart by thoracotomy, and was then processed through a 15-min centrifugation at 3,000 r/min. The mice feces were placed in sterile lyophilization tubes and stored in liquid nitrogen immediately before transferring to the −80°C refrigerator.

### 2.4. Enzyme-linked immunosorbent assay

Circulating amounts of TNF-α, IL-6, and IL-1β in each group were measured following the instructions of the corresponding ELISA test kit (MEIMIAN, Jiangsu, China).

### 2.5. 16S rDNA sequencing

Genomic DNA was extracted from fecal samples, and 1% agarose gel was used to monitor the DNA concentration and purity. After diluting the DNA to 1 ng/μL in sterile water, the V3-V4 region of the 16S rRNA gene was amplified for generating sequencing libraries by DNA PCR-Free Sample Preparation Kit (Illumina, United States). The sequence quality control was proceeded by means of Qubit@ 2.0 Fluorometer (Thermo) and Agilent Bioanalyzer system. In the final step, the sequence library was obtained through the Illumina platform.

### 2.6. Sequencing data analysis

Raw tags were acquired by stitching and filtering the reads of each sample to obtain clean tags (Caporaso et al., 2010; Magoc and Salzberg, 2011), which were further compared with the reference database (Edgar et al., 2011) for the removal of chimera sequences (Haas et al., 2011). Meaningful tags were obtained and further analysis was performed (Uparse software). Those sequences with more than 97% similarity were assigned to the same OTUs. The Silva Database and the Mothur algorithm were applied to annotate species. We managed to compute alpha and beta diversity. LDA score value at 4 was discarded in LEfSe analysis. To construct additional plots, we also applied the R software, which includes the following: rarefaction curves, PCoA plots, and differential plots highlighting group disparities.

### 2.7. Untargeted metabolomics analysis

Serum samples were kept at 4°C and vortexed. An appropriate amount of sample was accurately transferred to a tube with 400 μL methanol. After centrifuging for 10 min at 12,000 rpm at 4°C, the supernatant was then collected for subsequent concentration and dryness. We added a 150 μL of 2-chloro-l-phenylalanine with 80% methanol–water for redissolution. After removal, the supernatant was filtered through a 0.22 μm membrane and transferred into the detection bottle for LC–MS detection.

Maintained at 40°C, 0.25 mL/min flow rate, and 2 μL injection volume, the chromatography was applied by ACQUITY UPLC ^®^ HSS T3 (Waters, Milford, United States). Separation was conducted according to LC-ESI (+)-MS guidelines. As for another analysis, the analytes were carried out with acetonitrile and ammonium formate (5 mM). Instructions conducted separation.

Orbitrap Exploris 120 (Thermo, United States) was used for mass spectrometric detection of metabolites, with simultaneous MS1 and MS/MS acquisition. The parameters were standardized according to the manufacturer’s instructions.

### 2.8. Metabolomics data analysis

The raw data were first transformed into a uniform format using the ProteoWizard package (Smith et al., 2006). R XCMS package (Navarro-Reig et al., 2015) was then exploited for deep procession and integration. Identification of metabolites was made by accuracy mass (<30 ppm). All the MS/MS data were matched with HMDB, mass bank, LipidMaps, mzcloud, and KEGG. The LOESS signal correction method based on QC samples corrects and eliminates systematic errors. The substances with RSD > 30% in QC samples were filtered out from the data quality control. Moreover, we evaluated the robustness of the OPLS-DA model. The permutation test considered *p* value and VIP (projection of variable importance of OPLS – DA model) were chosen as the screening criteria. *p* < 0.05 and VIP > 1 were considered statistically significant. MetaboAnalyst then analyzed the metabolites for pathway analysis (Xia and Wishart, 2011). The identified metabolites in metabolomics were then mapped to the KEGG pathway. The KEGG Mapper tool assisted us in visualizing metabolites and pathways.

### 2.9. Statistical analysis

All statistical analysis proceeded with SPSS Statistics (IBM Software, United States) and figures were drawn using GraphPad Prism 7 (San Diego, California, United States). The discrepancy among groups was done using one-way ANOVA and Student–Newman–Keuls tests. Kaplan–Meier survival curve analysis was used to illustrate mortality between groups. *p* < 0.05 was considered statistically significant. All compared groups in this study at least included three independent samples.

## 3. Results

### 3.1. Characterization of main components of SFH by UPLC-MS analysis

**Figure 1:**
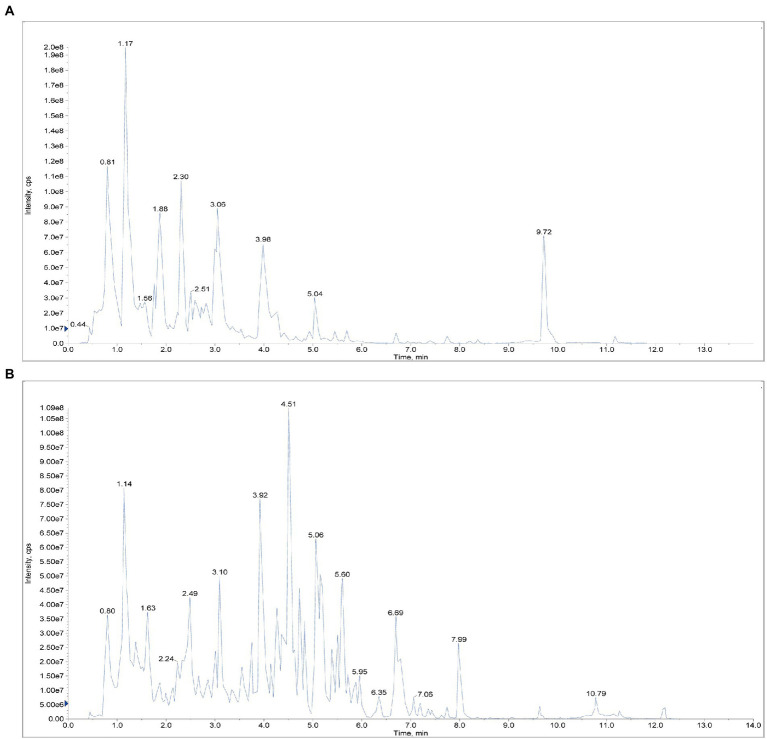
Positive and negative ion flow diagram. **(A)** Positive ion flow diagram. **(B)** Negative ion flow diagram.

The main compounds were identified in SFH decoction by UPLC-MS. As illustrated, the test results of positive and negative ion flow diagrams were shown (Figure 1). The left diagrams were shown as Supplementary Figure 1. 12 major compounds in SFH, including Rheic Acid, Catechin, Emodin, Gallic acid, Benzoylmesaconine, Ginsenoside Rg1, Chrysophanol, Aconitine, Aloe emodin, Mesaconitine, Ginsenoside Rb1, Ginsenoside Re were identified and characterized in Supplementary Figure 2.

### 3.2. SFH enhanced the survival rate and release of inflammatory cytokines in septic mice

**Figure 2:**
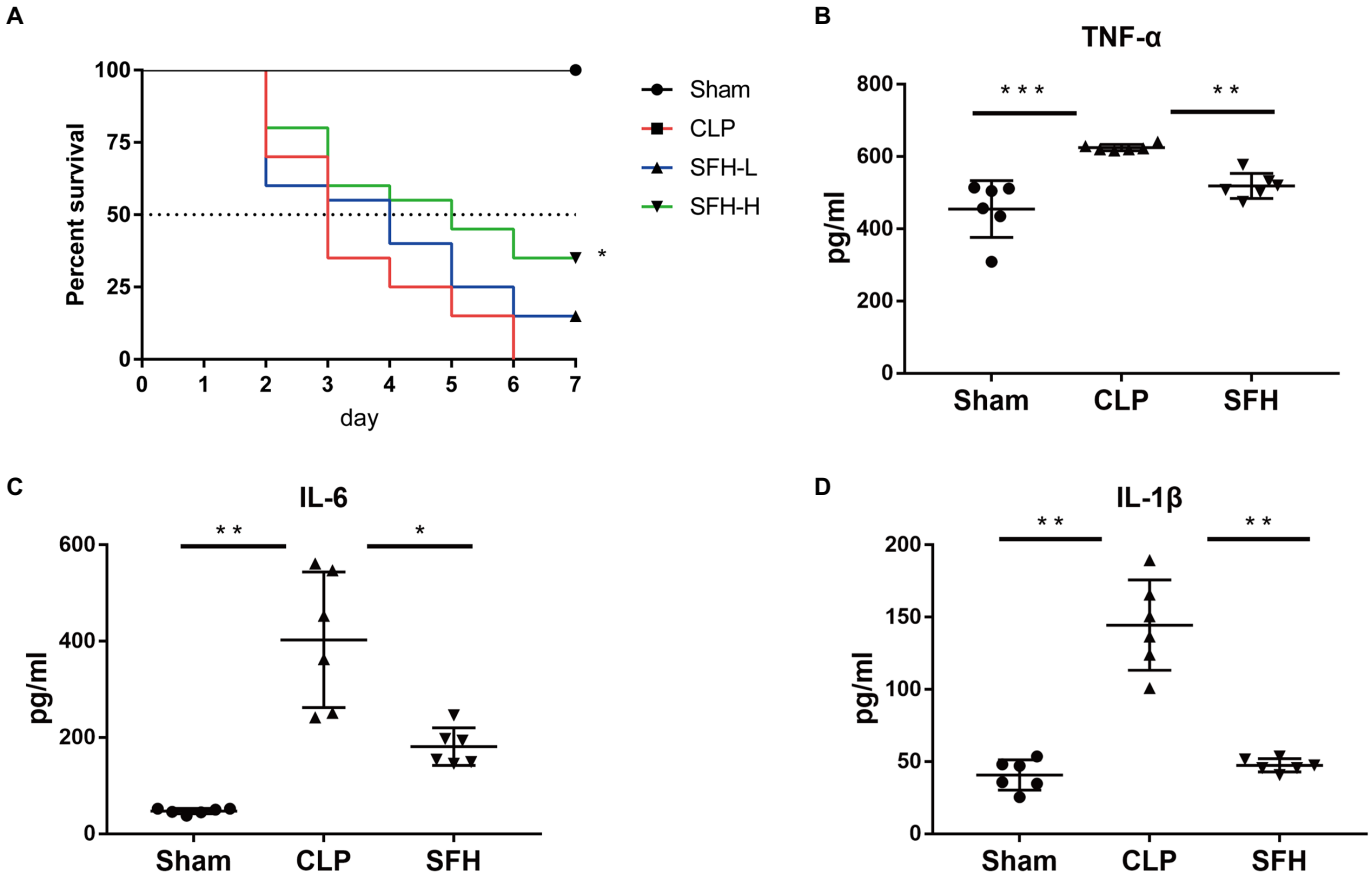
Effect of SFH on survival rate and the inflammatory cytokines expression of the septic mice. **(A)** Kaplan–Meier survival curve analysis of the survival rate. Sham group (*n* = 10), CLP, SFH-L, and SFH-H (24 h after operation, *n* = 20 per group) groups. **(B–D)** Expression of inflammatory cytokines in serum. Sham, CLP, and SFH (*n* = 6 per group) groups. Data were presented as means ± SD, *p < 0.05, **p < 0.01, ***p < 0.001.

First, we observed the dose effect of SFH in CLP-induced septic mice. As shown in Figure 2A, the survival rate, around 35% within 3 days and 0% within a week after CLP, was drastically reduced compared to the Sham mice (*p* < 0.05). Kaplan–Meier survival analysis demonstrated that a high dose of SFH could significantly prolong the 7-day survival rate to 35%, which was only 15% in the SFH-L group (*p* < 0.05, Figure 2A). Therefore, high-dose of SFH was chosen for the subsequent experiments. To further explore the mechanism of SFH in reducing mortality in sepsis, we also measured the levels of pro-inflammatory mediators in mice serum. In CLP-induced septic mice, circulating TNF-α, IL-6, and IL-1β were significantly increased compared with the Sham group mice. At the same time, SFH treatment drastically decreased the levels of these inflammatory cytokines (*p* < 0.05, Figures 2B–D).

### 3.3. The effects of SFH on the composition of gut microbiota in septic mice

**Figure 3:**
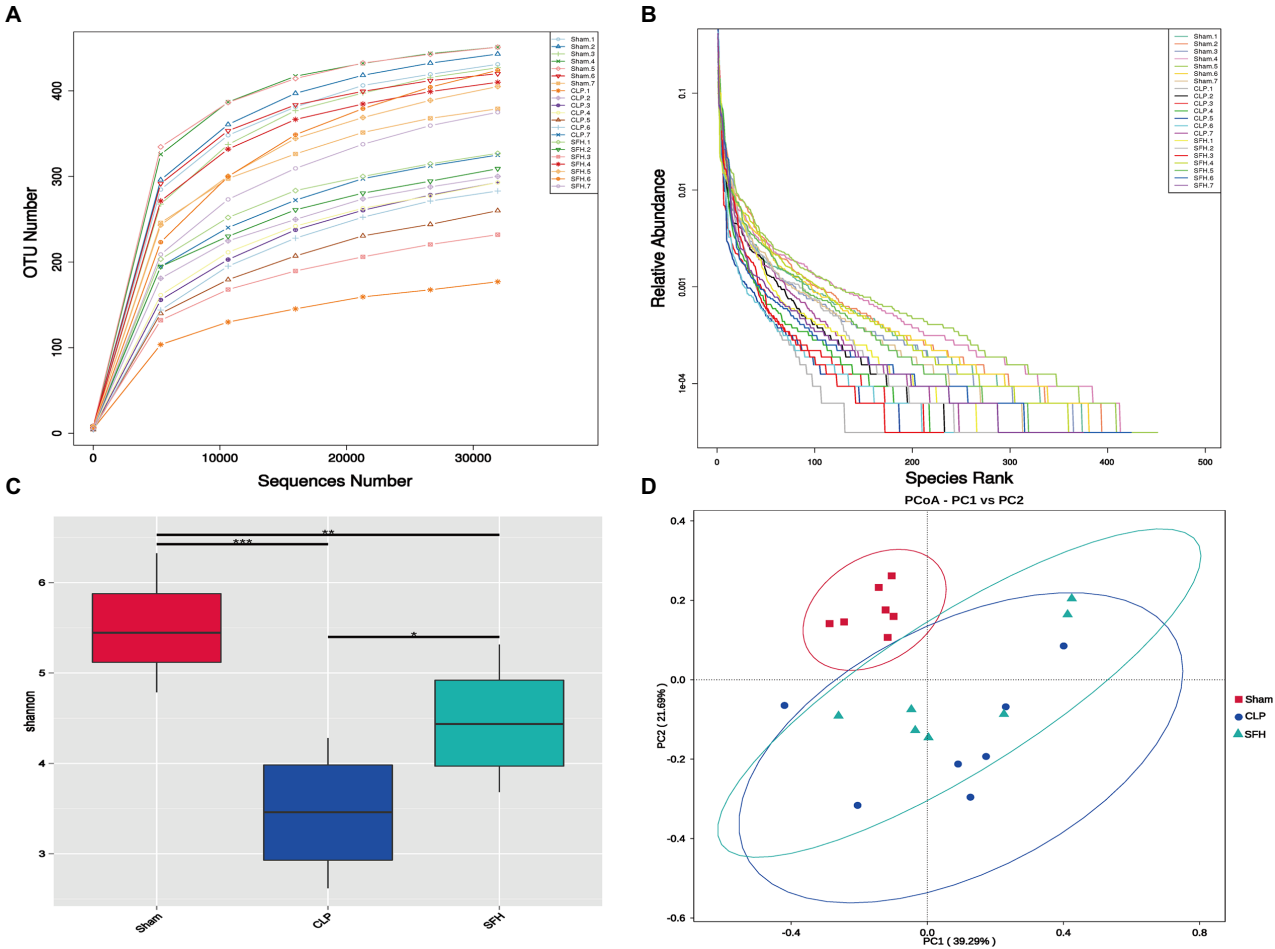
SFH treatment affected the gut microbiome diversity in septic mice. **(A)** Rarefaction curve and **(B)** rank abundance curve showing the plausibility of the sequencing data and indirectly reflecting species richness in the samples. **(C)** The Shannon index shows the alpha diversity in each group. **(D)** PCoA, based on weighted UniFrac distance, shows the beta diversity of each group (*n* = 7 per group). Data were presented as means ± SD, *p < 0.05, **p < 0.01, ***p < 0.001.

16S rDNA sequencing was used to depict the alterations in intestinal microbiome composition in SFH-treated septic mice. The rarefaction curve (Figure 3A) indicated that as the number of sequences rose, the curve flattened, indicating that the results of this sequencing were relatively reasonable. We also generated a rank abundance curve (Figure 3B), which corroborated the sequencing results. Changes in both diversity and richness of the gut flora were displayed by the Shannon index. The graphs indicated that the CLP could significantly lower the Shannon index in comparison to the Sham group (*p* < 0.001), whereas the low Shannon index could be restored by SFH induced by surgery (*p* < 0.05, Figure 3C). Moreover, PCoA results showed an apparent discrepancy in clusters among groups. When compared to the CLP group, the cluster of SFH was closer to the Sham group (Figure 3D). To conclude, these results indicated that CLP induced significant change in gut microbiota, and treatment with SFH significantly restored the disturbed gut microbiota in septic mice.

### 3.4. The effects of SFH on the abundance of bacterial flora in septic mice

**Figure 4:**
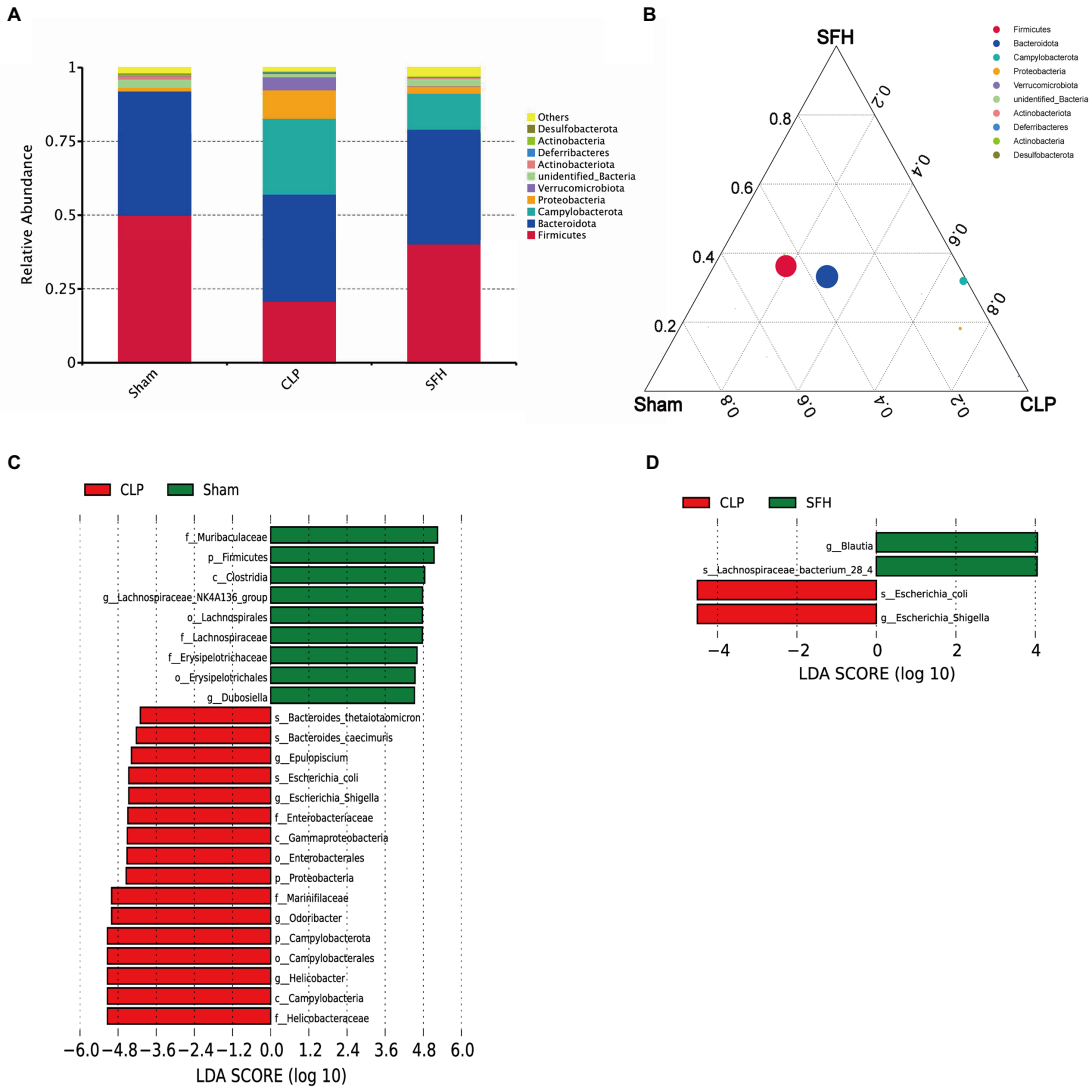
SFH changed the composition of gut microbiota in septic mice. **(A,B)** The phyla comparison among the groups of Sham, CLP, and SFH. **(C)** LEfSe analysis of Sham vs. CLP. **(D)** LEfSe analysis of CLP vs. SFH (*n* = 7 per group).

We then focused the study on investigating the effect of SFH on the bacterial abundance in septic mice. In each group, gut microbiota composition at the phylum level was displayed (Figures 4A,B). *Firmicutes* and *Bacteroides* represent the majority of intestinal flora in mice. The phyla of *Proteobacteria* and *Campylobacterota* were increased whereas *Firmicutes* was decreased in the CLP group. Interestingly, these alterations were reversed after the treatment of SFH. To identify the key phylotypes and biomarkers of gut microbiota among various groups, LEfSe analysis (LDA score > 4, *p* < 0.05) was performed. In the CLP group, the families of *Helicobacteraceae* and *Enterobacteriaceae*, including *Escherichia_coli* and *Escherichia_Shigella*, were abundant compared to the others (Figure 4C). However, treatment with SFH markedly increased the relative abundance of *Blautia* and *Lachnospiraceae bacterium_28_4* (Figure 4D).

### 3.5. The effects of SFH on serum metabolites in septic mice

**Figure 5:**
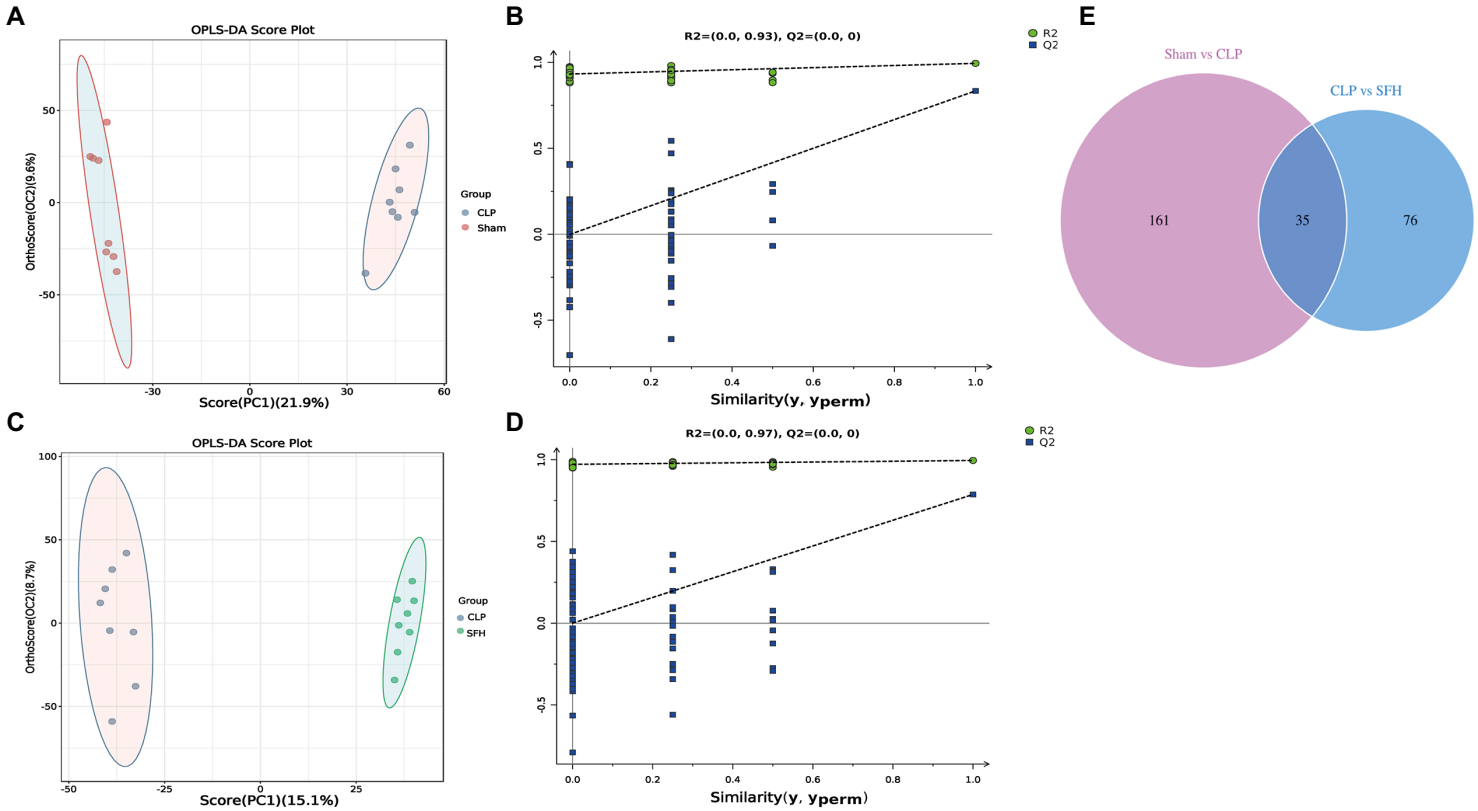
SFH treatment regulated the serum metabolites in septic mice. **(A,B)** Scores plots of OPLS−DA between the Sham and CLP groups and the corresponding coefficient of loading plots. **(C,D)** Scores plots of OPLS−DA between the CLP and SFH groups and the corresponding coefficient of loading plots. **(E)** Venn diagram of differential metabolites. Sham group vs. CLP group (purple) and CLP group vs. SFH group (blue) (*n* = 8 per group).

We further investigated the changes in serum metabolites after the septic mice were treated with SFH by metabolomics. OPLS-DA models were established based on metabolomics data in two ion modes from LC/MS. We found a considerable divergence between the CLP and SFH groups in both ion modes. In the positive ion mode, the Sham and CLP groups were well separated, indicating that sepsis significantly altered the mice metabolome (Figure 5A). Figure 5C also demonstrats that CLP and SFH groups were well separated, meaning that SFH significantly changed the metabolome of septic mice. The OPLS-DA substitution test plot fulfills any of the following points, showing that the findings are accurate and valid: the first point where all blue Q2 points are lower than the rightmost original blue Q2 point (the rightmost blue Q2 point in the plot is likely to overlap with the green R2 point in the top rightmost corner), the second point where the regression line crosses the horizontal coordinate or is less than 0. Therefore, the test results were reliable and valid (Figures 5B,D). A negative ion mode diagram is presented in Supplementary Figure 3. Moreover, metabolite expression was also different among the three groups. 196 critical metabolites were detected in the Sham and CLP groups, among which 146 were upregulated and the other 50 were downregulated. When comparing CLP and SFH groups, we found 111 different metabolites with statistical significance. More specifically, there were 54 upregulated and 57 downregulated metabolites (Table 1). By differential Venn diagram, 35 metabolites were commonly different (Figure 5E). The changes in differential metabolites are shown in Table 2. These results suggested that SFH restored the serum metabolites of sepsis mice similar to healthy mice.

**Table 1:** The statistical table on the number of differential metabolites.

Group name	Total sig metabolites	Upregulated	Downregulated
Sham vs. CLP	196	146	50
CLP vs. SFH	111	54	57

**Table 2:** The common differential metabolites in serum.

No	Formula	Metabolites	VIP		Trend		Pathway (CLP vs. SFH)
			Sham vs. CLP	CLP vs. SFH	Sham vs. CLP	CLP vs. SFH	
1	C3H4O4	Malonate	1.37	1.39	↑##	↓**	Pyrimidine metabolism
2	C7H9N	O-Toluidine	1.90	1.59	↑###	↑**	——
3	C4H4N2O2	Uracil	1.44	1.56	↑#	↓*	Pyrimidine metabolism
4	C4H4O5	Oxalacetic acid	1.28	1.29	↓#	↓*	Central carbon metabolism in cancer, Glucagon signaling pathway
5	C4H8O2S	3-Methylthiopropionic acid	1.62	1.79	↑###	↓***	——
6	C5H6N2O2	Imidazoleacetic acid	1.46	2.01	↑#	↓***	——
7	C5H7N3O	5-Methylcytosine	1.48	1.63	↑##	↓*	Pyrimidine metabolism
8	C8H9NO2	2-(Methylamino)benzoic acid	1.79	1.53	↑###	↑*	——
9	C7H6O3	4-Hydroxybenzoic acid	1.19	1.38	↑#	↑*	——
10	C5H6O5	Oxoglutaric acid	1.72	1.43	↑##	↓**	Central carbon metabolism in cancer, Glucagon signaling pathway
11	C7H6O4	Protocatechuic acid	1.40	1.87	↓#	↓***	——
12	C6H6N4O2	3-Methylxanthine	1.72	1.52	↑###	↓**	——
13	C7H11N3O2	1-Methylhistidine	1.61	1.33	↓##	↓*	——
14	C8H11NO3	Pyridoxine	1.53	1.47	↓##	↑*	——
15	C3H9O6P	Beta-Glycerophosphoric acid	1.42	1.07	↑###	↓*	——
16	C6H12O6	Allose	1.19	1.62	↑#	↓***	——
17	C13H10O	Benzophenone	1.73	1.40	↑###	↑*	——
18	C6H12O7	Gluconic acid	1.57	1.05	↑###	↓*	——
19	C8H11N3O3	N-Acetylhistidine	1.12	1.44	↑#	↓*	——
20	C11H12N2O2	L-Tryptophan	1.19	1.35	↑#	↓*	Central carbon metabolism in cancer, Glycine, serine and threonine metabolism, African trypanosomiasis
21	C9H11N5O2	Pyrimidodiazepine	1.37	1.61	↑#	↓*	——
22	C10H12N2O4	Hydroxykynurenine	1.54	1.63	↑###	↓**	——
23	C8H14N2O5S	gamma-Glutamylcysteine	1.32	1.18	↓#	↓*	——
24	C6H13O9P	Fructose 6-phosphate	1.61	1.26	↑##	↓*	Central carbon metabolism in cancer, Glucagon signaling pathway, Galactose metabolism
25	C10H11N5O4	5′-Dehydroadenosine	1.67	1.56	↑###	↑*	——
26	C16H22O4	Dibutyl phthalate	1.24	1.60	↑#	↓**	——
27	C18H28O2	Stearidonic acid	1.36	1.31	↑##	↑*	——
28	C18H32O3	9,10-Epoxyoctadecenoic acid	1.52	1.58	↑##	↑**	——
29	C20H40O2	Arachidic acid	1.60	1.70	↓##	↑**	——
30	C21H28O4	11-Dehydrocorticosterone	1.45	1.66	↓##	↑**	——
31	C20H32O4	Hepoxilin B3	1.38	1.72	↓#	↑**	Arachidonic acid metabolism
32	C22H42O2	Erucic acid	1.28	1.59	↑#	↑**	——
33	C6H14O12P2	Fructose 1,6-bisphosphate	1.59	1.86	↓##	↑**	Central carbon metabolism in cancer, Glucagon signaling pathway
34	C20H34O5	Troxilin B3	1.52	1.01	↑##	↓*	Arachidonic acid metabolism
35	C19H39N5O9	Antibiotic JI-20A	1.68	2.25	↓###	↓**	——

#*p* < 0.05 as compared to the CLP group; ##*p* < 0.01 as compared to the CLP group; ###*p* < 0.001 as compared to the CLP group; *p < 0.05 as compared to the SFH group; **p < 0.01 as compared to the SFH group; ***p < 0.001 as compared to the SFH group; ↑, content increased; ↓, content decreased; vs, versus; VIP, variable importance of projection. The *p*-values for the pathways listed in the table were less than 0.5.

### 3.6. The effects of SFH on the metabolic pathways in septic mice

**Figure 6:**
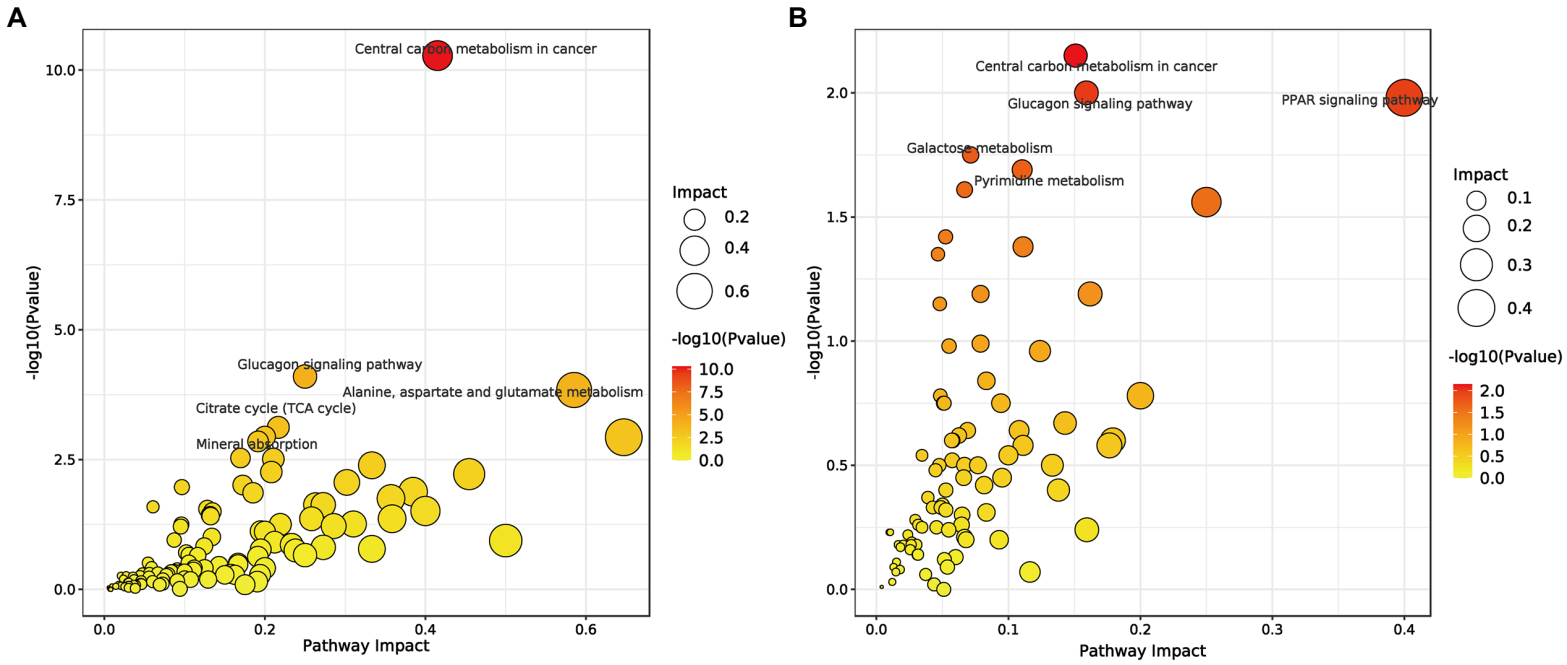
Metabolic pathways. **(A)** Summary of pathway analysis of serum samples between Sham and CLP groups. **(B)** Summary of pathway analysis of serum samples between CLP and SFH groups. Sham, CLP, and SFH groups (*n* = 8 per group).

Next, the critical metabolic pathways regulated by the Sham vs. CLP group and SFH vs. CLP were analyzed using MetPA software. Although sepsis-induced gut microbial imbalance is related with eight metabolic pathways, including central carbon metabolism in cancer, glucagon signaling, alanine, aspartate and glutamate metabolism, mineral absorption, TCA cycle, PPAR signaling pathway, galactose metabolism, and pyrimidine metabolism, SFH therapy can restore part of the metabolic balance. CLP surgery exerted an effect on the metabolic pathways including glucagon signaling, alanine, aspartate, glutamate metabolism, mineral absorption, and TCA cycle (Figure 6A), while SFH treatment probably influenced the central carbon metabolism, glucagon signaling, PPAR signaling pathway, galactose metabolism, and pyrimidine metabolism (Figure 6B).

### 3.7. Correlations between gut microbiota and serum metabolomics

**Figure 7:**
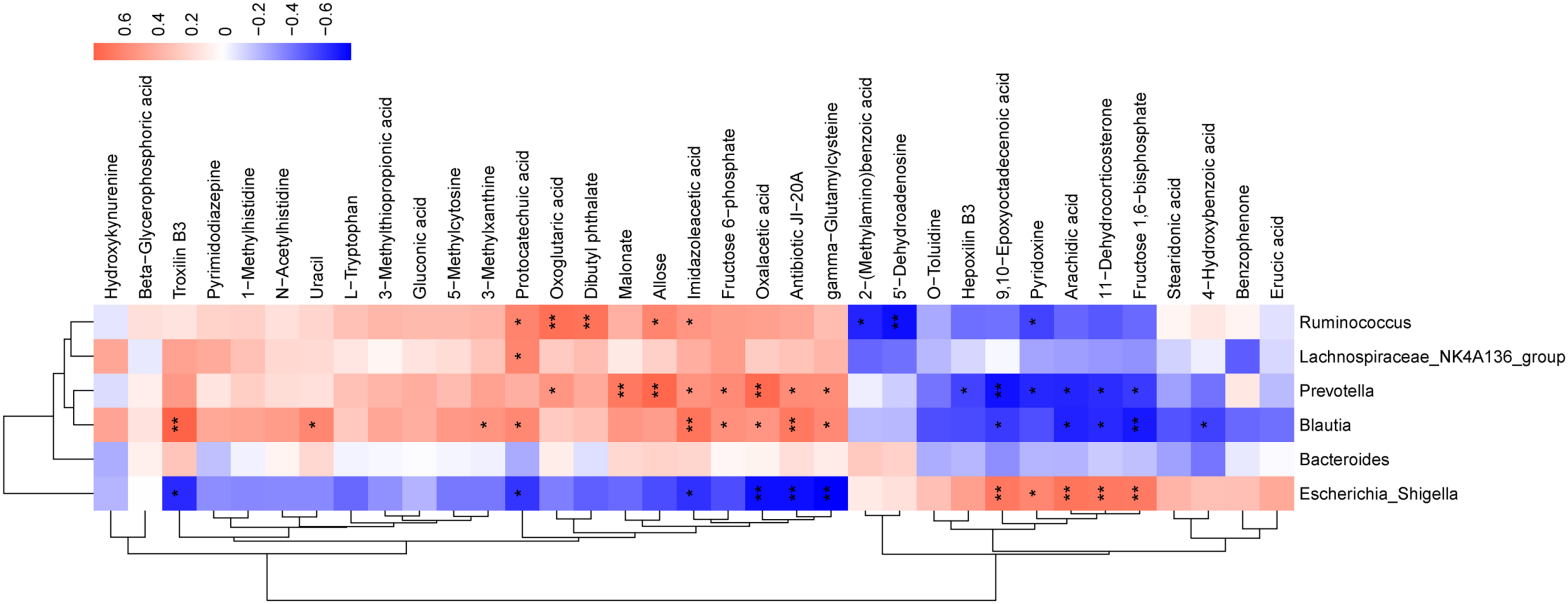
Correlation analysis of untargeted metabolomics and 16S rDNA sequencing. Red represents a positive correlation and blue represents a negative correlation. *p < 0.05, **p < 0.01, ***p < 0.001.

Spearman correlation was performed to investigate the relationship between differential serum metabolites and microbiome at the genus level in the treatment groups. As illustrated, *Bacteroides*, *Lachnospiraceae_NK4A136_group*, *Escherichia_Shigella*, *Blautia*, *Ruminococcus*, and *Prevotella* were related to most of the metabolites (Figure 7). The metabolites, including troxilin B3, protocatechuic acid, imidazoleacetic acid, oxalacetic acid, antibiotic JI-20A, and gamma−Glutamylcysteine, were positively linked with *Blautia* (*p* < 0.05) and inversely correlated with *Escherichia_Shigella* (*p* < 0.05). However, 9,10-Epoxyoctadecenoic acid, arachidic acid, 11-Dehydrocorticosterone, and fructose 1,6–bisphosphate demonstrated a negative correlation with *Blautia* (*p* < 0.05), but were positively correlated with *Escherichia_Shigella* (*p* < 0.05). Furthermore, *Prevotella* was positively correlated with oxoglutaric acid, malonate, allose, imidazoleacetic acid, fructose 6-phosphate, oxalacetic acid, antibiotic JI-20A, and gamma-Glutamylcysteine (*p* < 0.05), but negatively correlated with hepoxilin B3, 9,10-Epoxyoctadecenoic acid, pyridoxine, arachidic acid, 11-Dehydrocorticosterone, and fructose 1,6-bisphosphate (*p* < 0.05). *Lachnospiraceae_NK4A136_group* was positively correlated with protocatechuic acid (*p* < 0.05). *Ruminococcus* was positively associated with protocatechuic acid, oxoglutaric acid, dibutyl phthalate, allose, imidazoleacetic acid (*p* < 0.05), and negatively correlated with 2-(Methylamino) benzoic acid, 5′-Dehydroadenosine, and pyridoxine (*p* < 0.05). The results indicated that SFH could alter the composition of gut microorganisms and regulate certain metabolites *in vivo*.

## 4. Discussion

The high incidence and mortality rates have put sepsis as a main concern for researchers worldwide. Moreover, there is a lack of ideal treatment in clinical medicine. Antibiotics and supportive care are the mainstays of contemporary Western medicine therapy for sepsis (Varkouhi et al., 2020). Antibiotics, one of the most commonly used medicine for sepsis treatment, has the potential to modify the gut flora and hence have a detrimental influence on sepsis outcomes (Dethlefsen et al., 2008; Adelman et al., 2020). In the clinical practice of sepsis, TCM, such as XueBiJing injection (XBJ), ShenFu injection, and ShengMai formula, are also powerful alternatives (Liu L. W. et al., 2020). The application of TCM places a strong emphasis on using herbal formulations to regulate immune responses to illness, such as clearing heat and toxin, facilitating blood circulation, enhancing gastrointestinal function, and strengthening body resistance (Fan et al., 2020). Herbal medicine is capable of regulating both intestinal flora composition and metabolism (Cheng and Yu, 2021). In the management of sepsis, numerous clinical study meta-analyses have demonstrated the necessity of herbal medicine (Liang et al., 2015; Wen et al., 2021). Since TCM can improve intestinal microbiota disorders and maintain intestinal homeostasis. In this study, we demonstrated that SFH improved 7-day survival in septic mice by concurrently inhibiting pro-inflammatory TNF-α, IL-6, and IL-1β cytokines. We are the first to elucidate the protective mechanism of SFH in treating sepsis. The remission of sepsis with SFH-treatment might be attributed to gut microbiota alteration *via* multiple metabolic pathway regulation.

Designed to treat septic syndrome and COVID-19 patients, SFH was composed of 49 active compounds, namely emodin, rhein, aconitine, ginsenoside Rb1, etc. (Liu T. et al., 2020). Also, the study reported that rheic acid, catechin, emodin, gallic acid, benzoylmesaconine, ginsenoside Rg1, chrysophanol, aconitine, aloe emodin, mesaconitine, ginsenoside Rb1, and ginsenoside Re were detected in SFH. With multi-component, multitarget, and multi-channel systems, Chinese herbs have unique and sophisticated mechanisms in regulating the immune response (Usmani et al., 2021). For instance, rhein has been shown to inhibit the production of pro-inflammatory cytokines (Wu et al., 2020). Previous researches have confirmed that emodin could alleviate sepsis-induced intestinal damage and suppress inflammatory responses (Chen et al., 2016; Shang et al., 2021). Ginsenoside Rg1 can also improve the survival rate of septic mice by modulating the immune response and sepsis-induced lung injury (Zou et al., 2013; Wang et al., 2019). Aconitine alleviates myocardial injury by improving mitochondrial function (Wang et al., 2021). The compositional complexity of TCMs is fundamental to achieving the multi-target action mode of TCMs, while it can hamper the mechanistic understanding of their therapeutic benefits. Small molecules and polysaccharides are two essential and dominant chemical types in TCMs decoctions. Investigations have been made that the functions of polysaccharides were underestimated due to poor absorption and ambiguous mechanisms. In immune-deficiency mice, polysaccharides upregulated the number of peripheral NK cells as well as enhanced the release of cytotoxic perforin and granzyme, which could strengthen the lytic ability of NK cells cytotoxicity (Sun et al., 2016). Yoo and his colleagues examined the immunomodulatory traits of Panax ginseng polysaccharide (GP) in the setting of influenza (Yoo et al., 2012). The main compounds identified in SFH decoction included rheic Acid, Catechin, Emodin, Gallic acid, Benzoylmesaconine, Ginsenoside Rg1, Ginsenoside Re, Aconitine, etc. Here, we unraveled the effects and made assumptions about the mechanisms of SFH treatment in sepsis. Our group has previously reported the relationship between Emodin and intestinal barrier, revealing that Emodin might be a pivotal component of SFH (Zhang et al., 2022). We have also conducted studies on Ginsenoside Rg1, Rb1, and Re for the treatment of sepsis, the results of which are yet to be published. Our research showed that SFH could reduce the inflammatory response caused by sepsis. Therefore, SFH may arise as a promising candidate for treating sepsis.

The gut has long been characterized as the motor of multiple organ dysfunction syndromes (MODS; Mittal and Coopersmith, 2014). Maintaining enteric and systemic immunological homeostasis depends on a balanced microbiota, and alteration of the intestinal microbiota’s integrity may make people more susceptible to sepsis (Haak and Wiersinga, 2017). A novel focus for the therapy of sepsis may be the regulation of intestinal flora (Haak et al., 2018). Patients who are critically ill may have dysbiosis, with a reduction in “health-promoting” commensal flora (such as *Firmicutes* or *Bacteroidetes*) and accompanied by an enrichment in potentially pathogenic gut bacteria (such as *Proteobacteria*) (Wozniak et al., 2022). Prevalence in *Proteobacteria* is recognized as a potential diagnostic signature of disease (Shin et al., 2015). Gut microbiota can also bar the invading microbes from gastrointestinal tract colonization, also known as “colonization resistance” (Kim et al., 2017). Studies have pointed out that *Escherichia_coli*, *Proteus*, and *Enterobacter* might result in occurrence of bacteremia in debilitated patients since enteric bacilli translocate more efficiently, especially obligate anaerobes (Steffen et al., 1988). Our study showed a significant difference in gut microbial composition between the Sham and CLP groups. As expected, SFH could modulate the abundance of *Escherichia_Shigella*, *Escherichia_coli*, *Blautia*, and other bacteria, restoring them to levels similar to those of healthy mice. As *Escherichia_Shigella* has the ability to invade and destruct human colonic epithelium (Belotserkovsky and Sansonetti, 2018), it may incur sepsis-related neural inflammation (Zhao et al., 2022). *Blautia*, an anaerobic bacteria with probiotic traits, plays specific roles in metabolic diseases, inflammatory settings, and bio-transformation (Liu et al., 2021). As discussed above, SFH can regulate the intestinal microbiota of septic mice by increasing beneficial bacteria and reducing pathogenic bacteria.

Metabolomics can provide evidence of metabolite-concentration changes, while metabolic alterations further reflect and reveals disease-related biomarkers or potential mechanisms (Ingels et al., 2018; Jiang et al., 2019). Animal studies suggested that changes in serum metabolic profiles took place earlier than organ dysfunction (Xu et al., 2008) and plasma metabolism alteration serve as a hallmark of sepsis (Beloborodova et al., 2018). Our study showed 35 common differential metabolites in the serum of all groups, including L-tryptophan, uracil, gluconic acid, protocatechuic acid, and gamma-Glutamylcysteine. Gluconic acid and uracil were characteristic metabolites identified in models of heat injury and/or sepsis (Liu et al., 2010). The utilization of uracil favors the diagnosis of multiple traumas complicated with sepsis (Feng et al., 2022). Animal experiments showed that additional provision of L-tryptophan protected mice from lipopolysaccharide (LPS)-induced acute lung injury (Liu S. et al., 2020). Protocatechuic acid attenuates LPS-induced septic lung injury in mice (Alsharif et al., 2021). In mice, gamma-Glutamylcysteine restored systemic inflammatory responses and thus attenuated sepsis lethality (Yang et al., 2019). These results suggested that SFH could reveal a protective effect in septic mice by regulating these metabolites. The results of untargeted metabolomics in serum strongly evidenced that SFH modulated the glucagon signaling pathway, PPAR signaling pathway, galactose metabolism, and pyrimidine metabolism. Such as previous studies have shown that regulation of PPAR signaling can inhibit pro-inflammatory cytokine production and prevent inflammatory derangements in sepsis (Wang et al., 2017; Iwaki et al., 2019). Galactosemia being a treatable metabolic disorder, infants with galactosemia are at higher risk for *Escherichia_coli* neonatal sepsis (Rathi and Rathi, 2011). Intestinal flora can affect the host’s health by regulating metabolism (Ursell et al., 2014). Spearman correlation analysis showed *Bacteroides*, *Lachnospiraceae_NK4A136_group*, *Escherichia_Shigella*, *Blautia*, *Ruminococcus*, and *Prevotella* showed correlations with most of the metabolites such as L-tryptophan, uracil, gluconic acid, protocatechuic acid, gamma-Glutamylcysteine. These metabolites were associated with multiple metabolic pathways. Our study indicated that SFH can alter the intestinal flora in septic mice, thereby regulating metabolites.

There are still some limitations in our study. We performed serum composition of SFH after administration, but the assessment of the function of the specific components in septic mice still needs further investigation. To dissect the clinical translation of SFH in the administration of sepsis, an enlarge in the sample size of *in vivo* animal models and more detailed molecular biology experiments are required.

## 5. Conclusion

In conclusion, our study demonstrated that SFH is a promising strategy for treating sepsis by reducing mortality and suppressing the inflammatory response. Moreover, our results revealed that the mechanism of SFH for treating sepsis is related to the improvement of gut microbiota dysbiosis and modulation of the Glucagon signaling pathway, PPAR signaling pathway, Galactose metabolism, and Pyrimidine metabolism. This is the first study to investigate the effect of SFH on the gut microbiome and metabolism in septic mice, which may provide novel insights into the clinical application of SFH in sepsis.

## Data availability statement

The datasets presented in this study can be found in online repositories. The names of the repository/repositories and accession number(s) can be found at: https://www.ncbi.nlm.nih.gov/, PRJNA905565.

## Ethics statement

The animal study was reviewed and approved by the Animal Care and Use Committee of Beijing Institute of Chinese Medicine.

## Author contributions

QL and SH designed the research. SH, YG, and JZ completed experimental quality control. SH, LL, XX, and NW directed the experiment. SH, CZ, YH, BL, and HY performed the experiment and analyzed the data. SH, CZ, and LL wrote the manuscript. All authors contributed to the article and approved the submitted version.

## Funding

This work was supported by grants from the National Natural Science Foundation of China (82174157, 81803879, and 82274283), and the National Multidisciplinary Innovation team project of traditional Chinese medicine (ZYYCXTD-D-202201).

## Conflict of interest

The authors declare that the research was conducted in the absence of any commercial or financial relationships that could be construed as a potential conflict of interest.

## Publisher’s note

All claims expressed in this article are solely those of the authors and do not necessarily represent those of their affiliated organizations, or those of the publisher, the editors and the reviewers. Any product that may be evaluated in this article, or claim that may be made by its manufacturer, is not guaranteed or endorsed by the publisher.
