# Supplementary material for: Alterations in the gut microbiome and metabolome profiles of septic mice treated with Shen FuHuang formula
Source: Front Microbiol. 2023 Mar 8;14:1111962. doi: 10.3389/fmicb.2023.1111962 (PMC10030955; doi:10.3389/fmicb.2023.1111962)
Supplement: Supplementary file 1 [file Data_Sheet_1.docx]

Supplementary Material

Alterations in the gut microbiome and metabolome profiles of septic mice treated with Shen Fuhuang Formula

**Shasha He^†^, Chunxia Zhao^†^, Yuhong Guo, Jingxia Zhao, Xiaolong Xu, Yahui Hu, Bo Lian, Haoran Ye, Ning Wang, Lianxiang Luo*, and Qingquan Liu***

*** Correspondence:** Corresponding Author: Qingquan Liu; liuqingquan_2003@126.com

Lianxiang Luo; Luolianxiang321@gdmu.edu.cn

Shasha He ^†^ and Chunxia Zhao ^†^ These authors have contributed equally to this work.

## Supplementary Figures


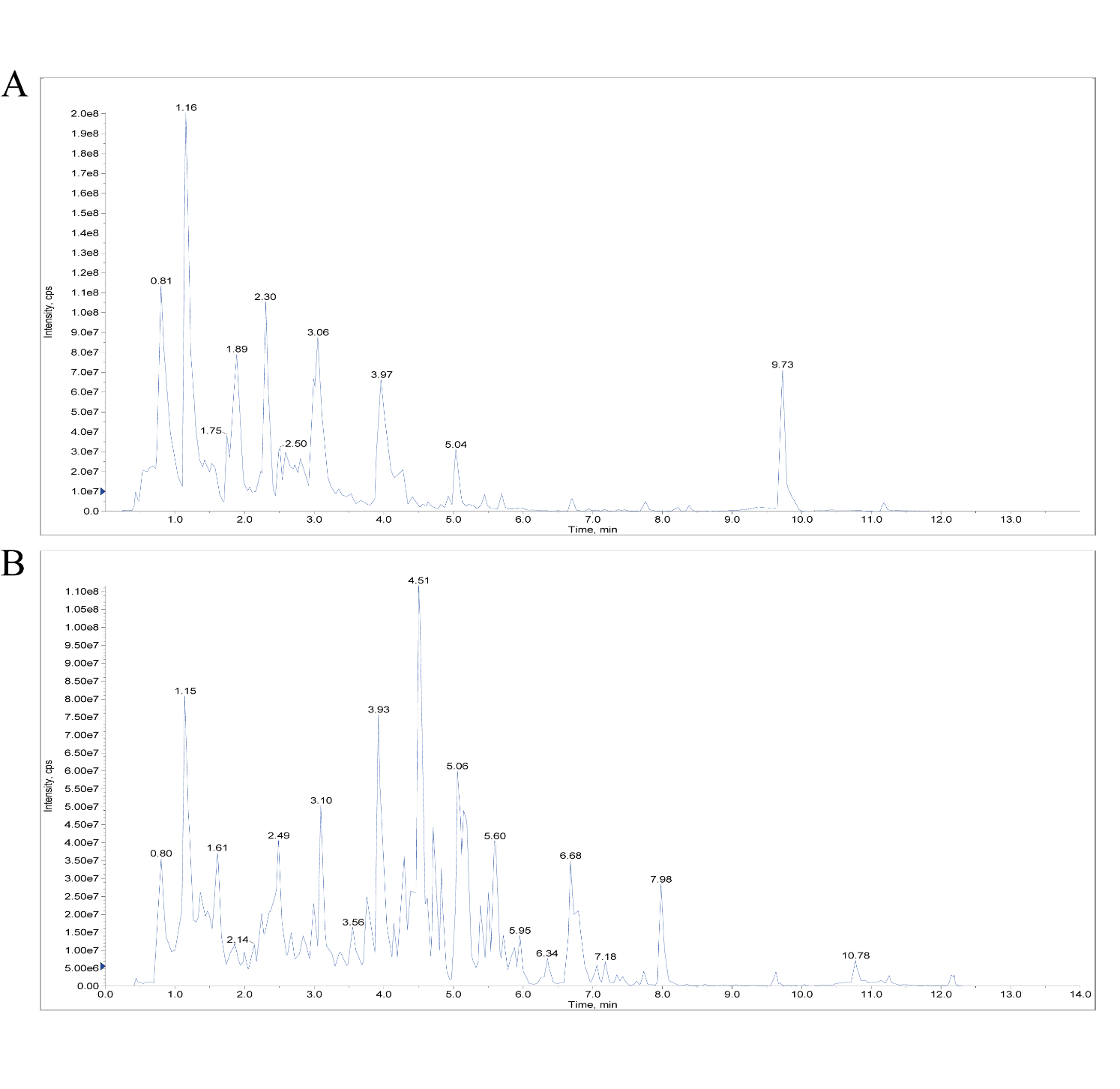


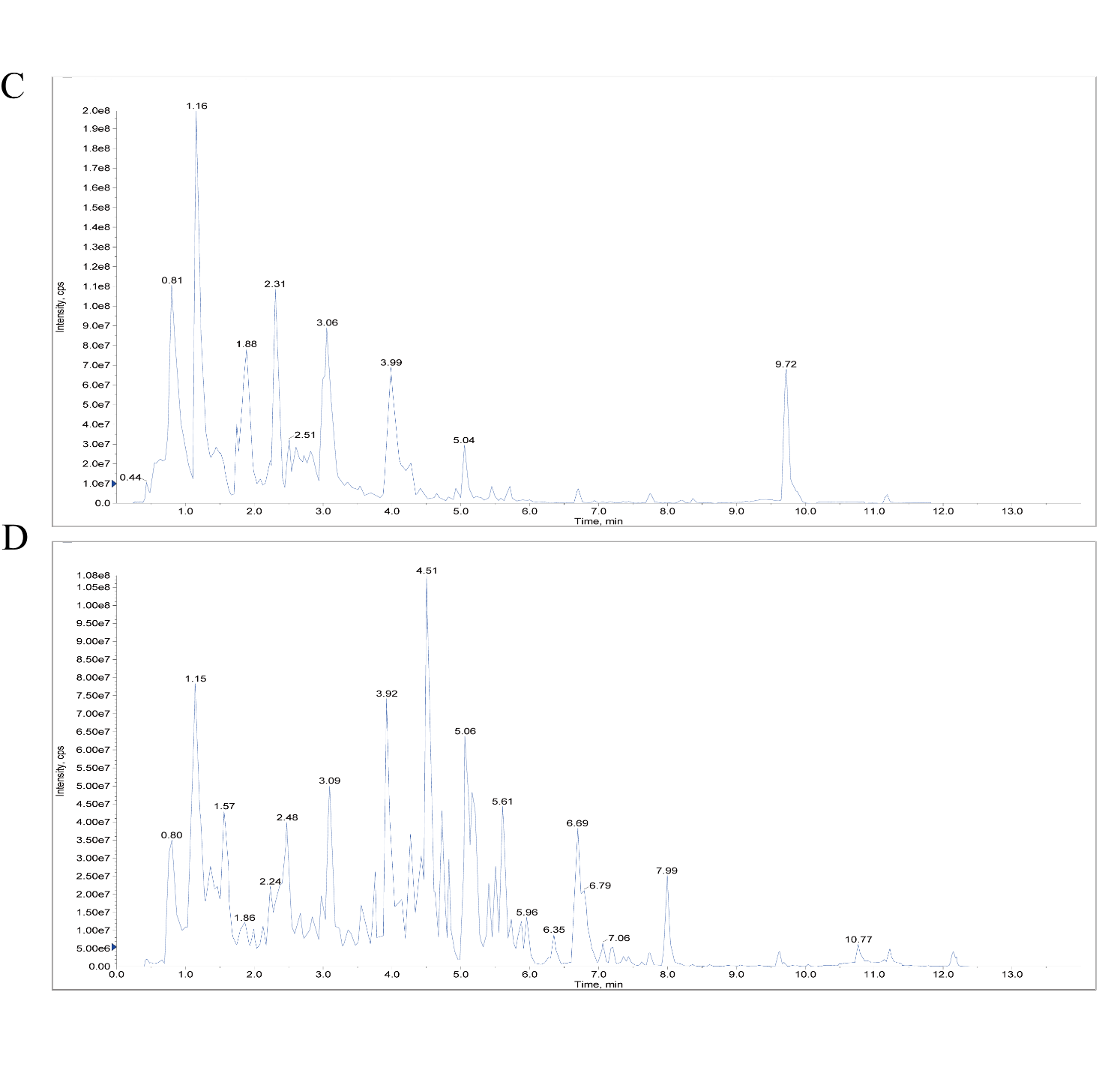


## Supplementary Figure 1. Positive and negative ion flow diagram. (A, C) positive ion flow diagram, (B, D) negative ion flow diagram.


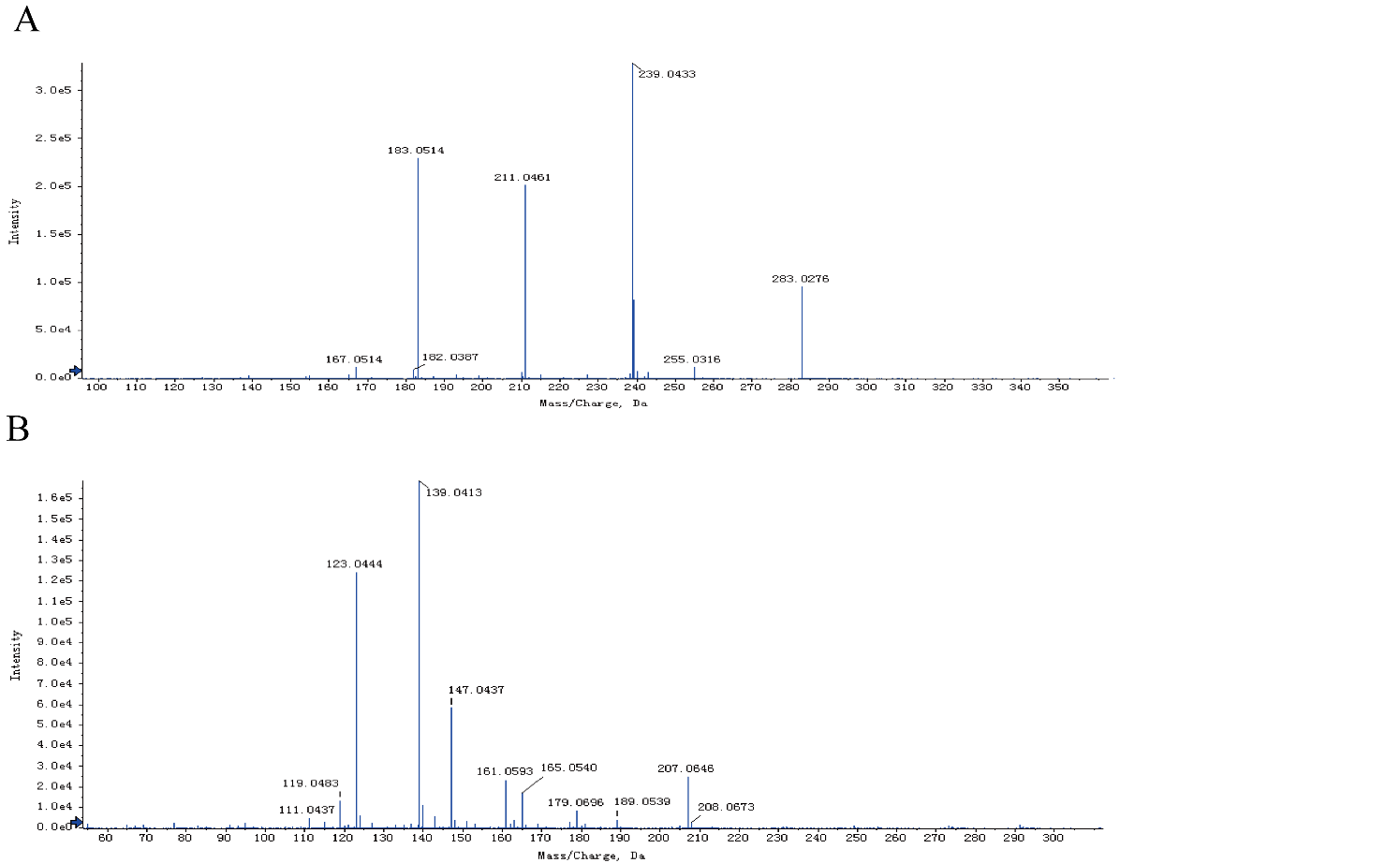


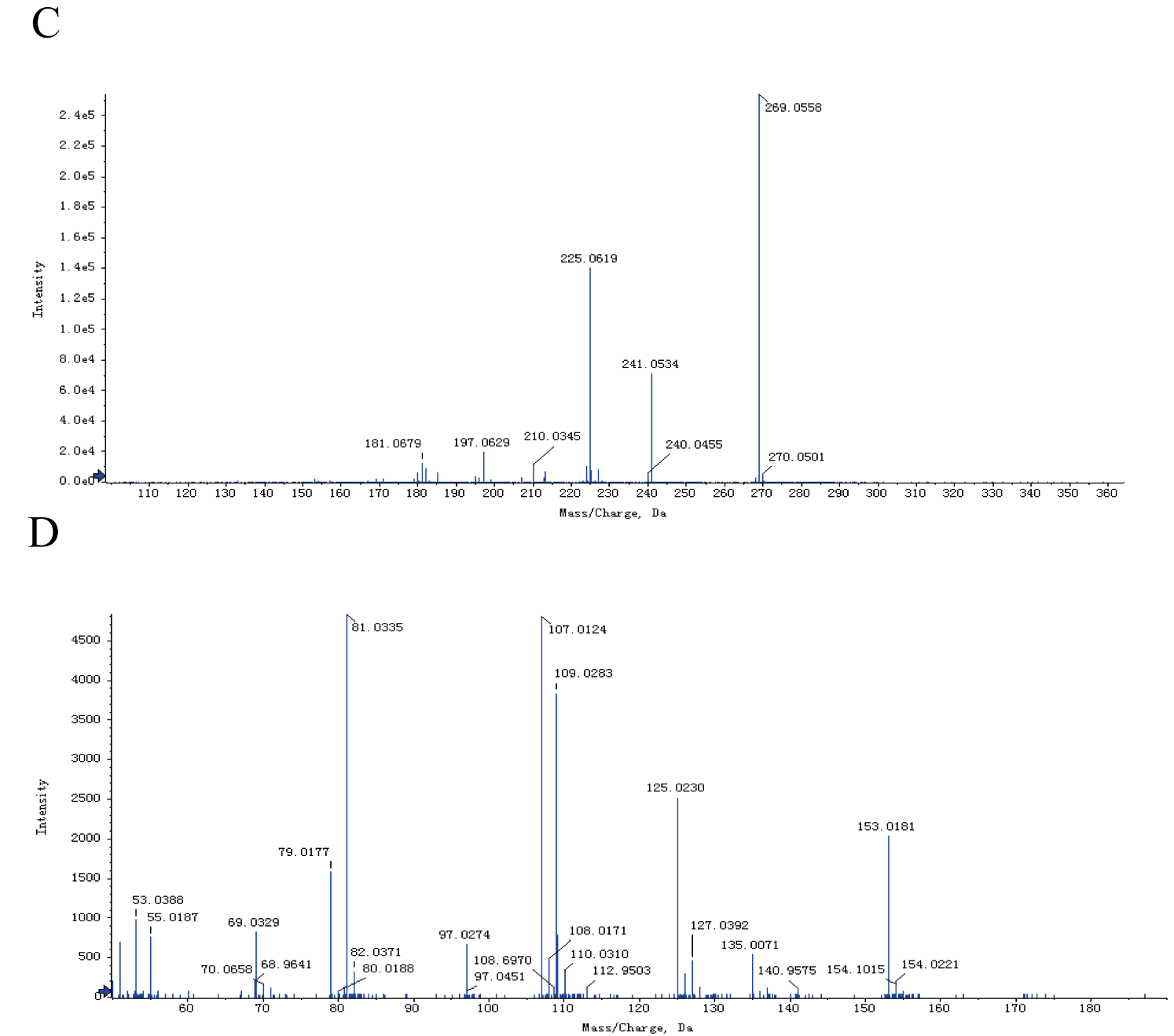


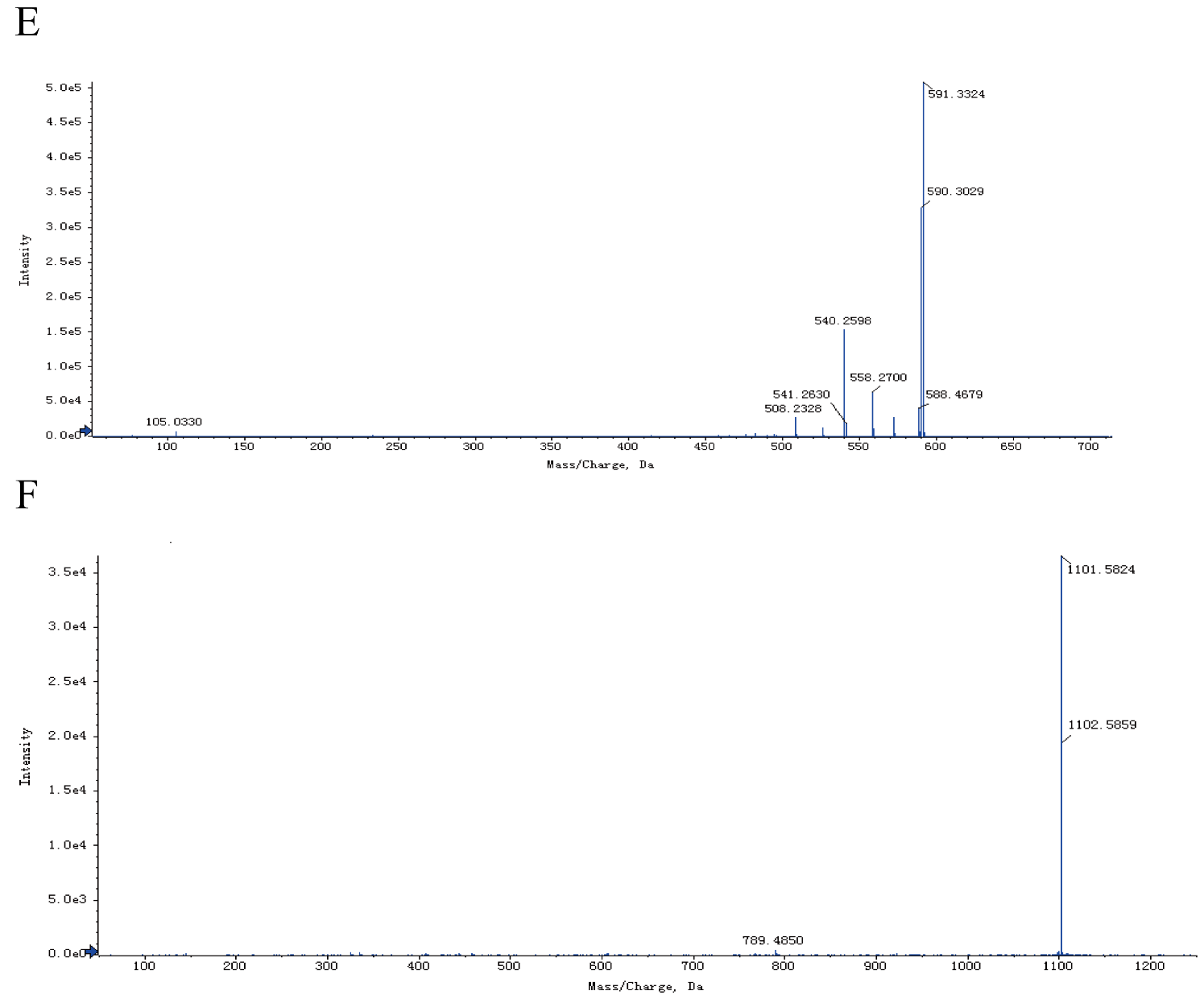


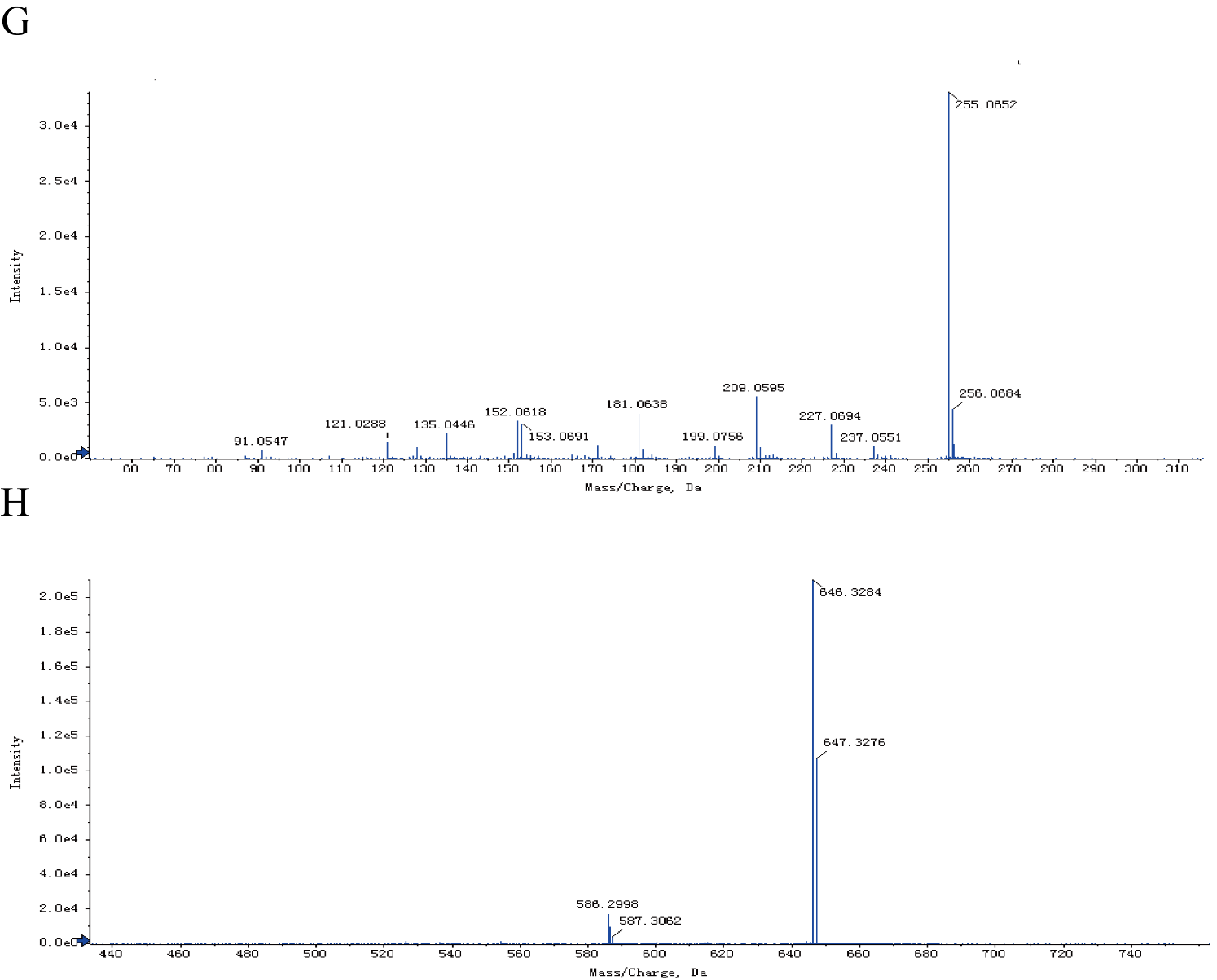


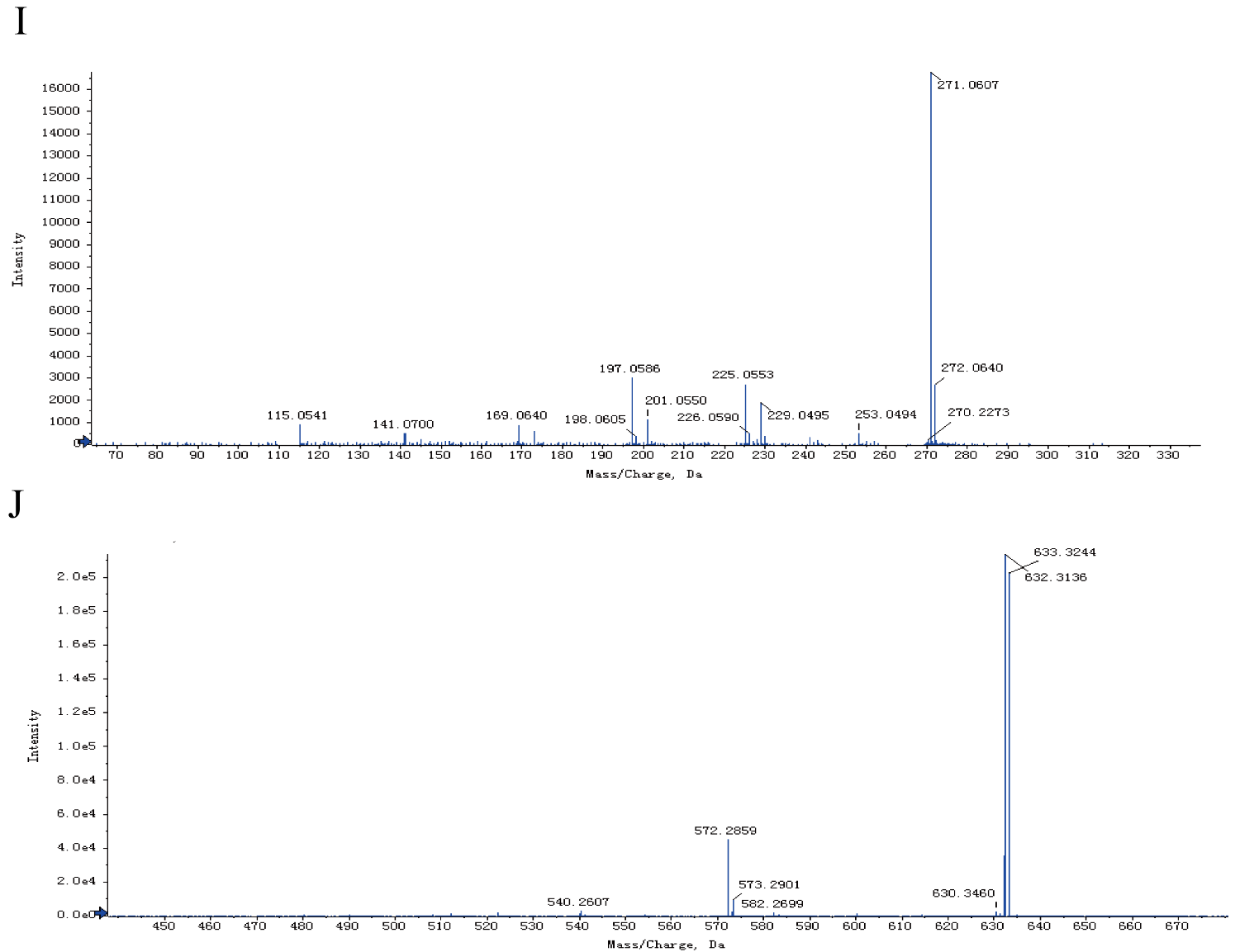


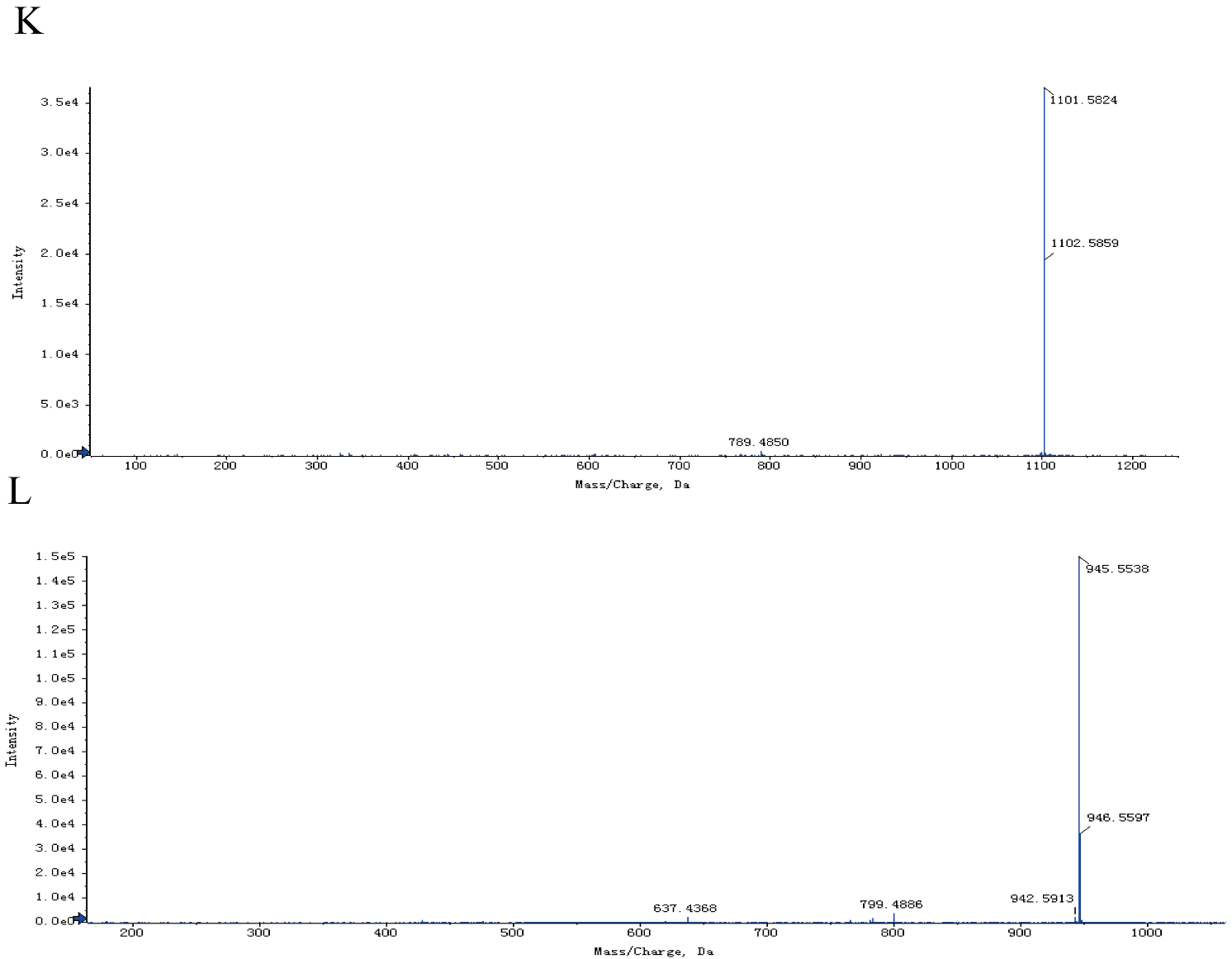


## Supplementary Figure 2. Major compounds in SFH. (A) Rheic Acid (B) Catechin (C)Emodin (D)Gallic acid (E) Benzoylmesaconine (F) Ginsenoside Rg1(G) Chrysophanol (H) Aconitine (I) Aloe emodin (J) Mesaconitine (K) Ginsenoside Rb1 (L) Ginsenoside Re


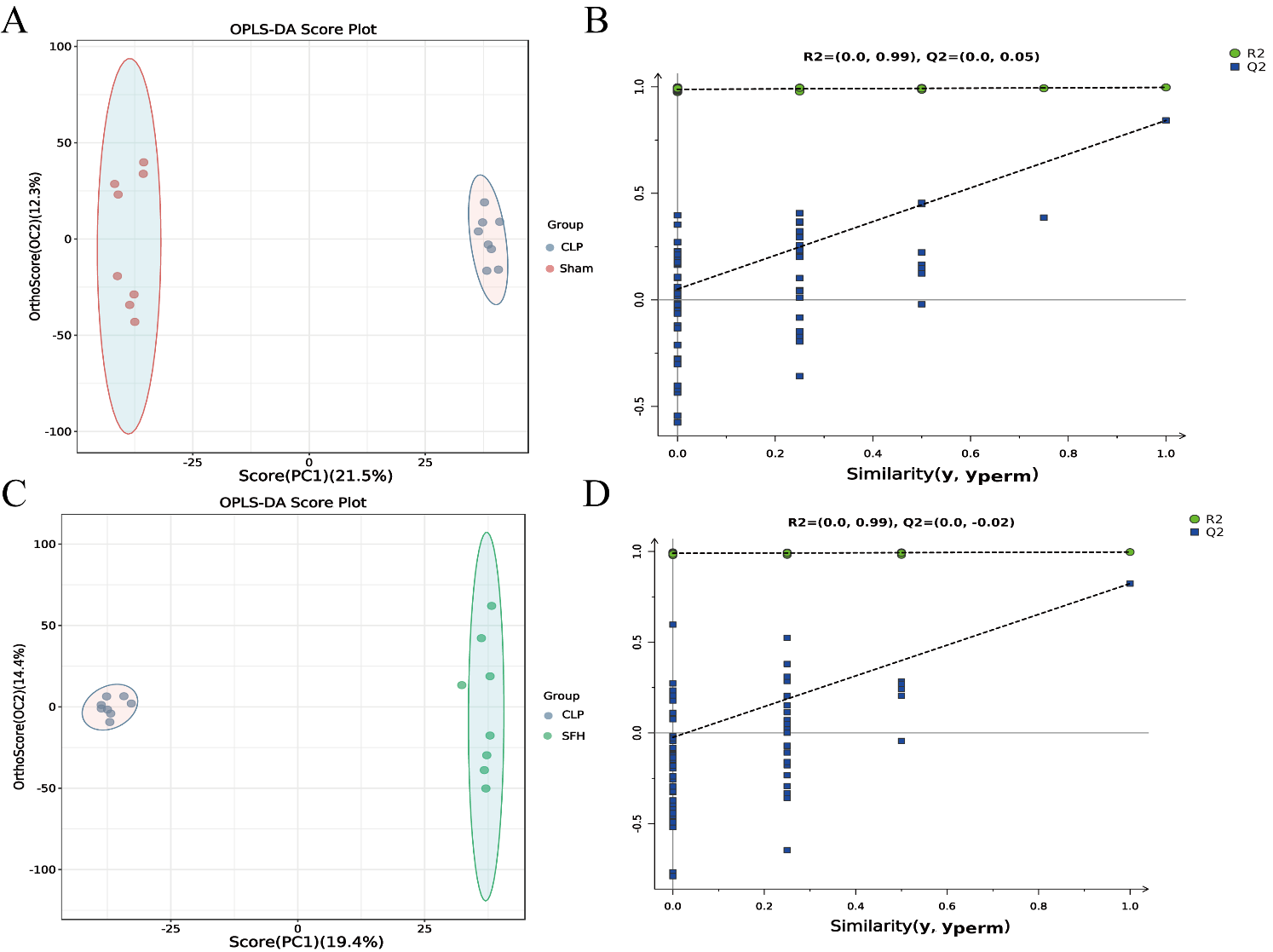


**Supplementary Figure 3.** Negative ion modes (A, B) Scores plots of OPLS−DA between the Sham and CLP groups and the corresponding coefficient of loading plots. (C, D) Scores plots of OPLS−DA between the CLP and SFH groups and the corresponding coefficient of loading plots.
